# Sivelestat Alleviates Atherosclerosis by Improving Intestinal Barrier Function and Reducing Endotoxemia

**DOI:** 10.3389/fphar.2022.838688

**Published:** 2022-04-04

**Authors:** Hezhongrong Nie, Qingquan Xiong, Guanghui Lan, Chunli Song, Xiaohong Yu, Lei Chen, Daming Wang, Tingyu Ren, Zeyan Chen, Xintong Liu, Yiwen Zhou

**Affiliations:** ^1^ Center of Clinical Laboratory, Shenzhen Hospital, Southern Medical University, Shenzhen, China; ^2^ Department of General Surgery, Shenzhen Hospital, Southern Medical University, Shenzhen, China

**Keywords:** atherosclerosis, sivelestat, intestinal permeability, endotoxemia, inflammation

## Abstract

Emerging evidence suggests that atherosclerosis, one of the leading phenotypes of cardiovascular diseases, is a chronic inflammatory disease. During the atherosclerotic process, immune cells play critical roles in vascular inflammation and plaque formation. Meanwhile, gastrointestinal disorder is considered a risk factor in mediating the atherosclerotic process. The present study aimed to utilize sivelestat, a selective inhibitor of neutrophil elastase, to investigate its pharmacological benefits on atherosclerosis and disclose the gastrointestinal–vascular interaction. The activation of intestinal neutrophil was increased during atherosclerotic development in Western diet-fed ApoE^-/-^ mice. Administration of sivelestat attenuated atherosclerotic phenotypes, including decreasing toxic lipid accumulation, vascular monocyte infiltration, and inflammatory cytokines. Sivelestat decreased intestinal permeability and endotoxemia in atherosclerotic mice. Mechanistically, sivelestat upregulated the expression of zonula occludens-1 in the atherosclerotic mice and recombinant neutrophil elastase protein-treated intestinal epithelial cells. Meanwhile, treatment of sivelestat suppressed the intestinal expression of inflammatory cytokines and NF-κB activity. In contrast, administration of lipopolysaccharides abolished the anti-atherosclerotic benefits of sivelestat in the Western diet-fed ApoE^-/-^ mice. Further clinical correlation study showed that the circulating endotoxin level and intestinal neutrophil elastase activity were positively correlated with carotid intima-medial thickness in recruited subjects. In conclusion, sivelestat had pharmacological applications in protection against atherosclerosis, and intestinal homeostasis played one of the critical roles in atherosclerotic development.

## Introduction

Atherosclerosis, one of the major phenotypes of cardiovascular diseases (CVDs), is the leading cause of cardiovascular morbidity and mortality (Libby et al., 2016). It is associated with increased vascular inflammation and plaque formation. Recently, emerging studies have disclosed the potential crosstalk between the cardiovascular system and gastrointestinal homeostasis (Cani et al., 2007; Pendyala et al., 2012). In patients with atherosclerosis, there was obvious induction of plasma endotoxin activity, which was accompanied by intestinal injuries (Pendyala et al., 2012). Administration of intestinal toxic lipopolysaccharides (LPS) accelerated vascular inflammation and atherosclerotic plaque formation by eliminating anti-atherosclerotic benefits of healthy intestinal bacteria in apolipoprotein (Apo) E-deficient mice (Jin Li et al., 2016). Trimethylamine N-oxide (TMAO), a product of intestinal microorganisms, could promote the atherosclerosis process and accelerate the pathological process of cerebrovascular diseases (Wang et al., 2015). Apo A-I, synthesized in small intestinal cells, was a potential target for protection against atherosclerosis (Chen et al., 2020). Therefore, targeting intestinal homeostasis has therapeutic benefits in combating atherosclerosis.

Structural disorders of the gastrointestinal system mainly involve disruption of intestinal permeability, leakage of toxic substances into circulation, and a consequent inflammatory response (Andreasen et al., 2008). In patients with cardiovascular diseases, the intestinal barrier was disrupted, whereas circulating toxic factors were remarkably upregulated (Kim et al., 2018; Noval Rivas et al., 2019). Western diet-induced atherosclerotic mice exhibited abnormal intestinal permeability, but improvement of intestinal permeability could alleviate diet-induced intestinal disorders and atherosclerotic plaque formation (Jin Li et al., 2016; Zhu et al., 2018). Mechanistic studies identified intestinal epithelial zonulin proteins, majorly controlled by zonula occludens-1 (ZO-1), and determined the intestinal permeability (Fasano, 2011; Carrera-Bastos et al., 2018). The circulating level of zonulin was closely associated with plasma endotoxin concentrations in patients with myocardial infarction (Carrera-Bastos et al., 2018). Western diet-induced atherosclerotic mice had a lower expression of intestinal ZO-1 but severe endotoxemia (Jin Li et al., 2016). Meanwhile, the biogenesis and activity of intestinal epithelial ZO-1 were dampened by the local intestinal and systemic inflammation. In patients with immune diseases, the tight junction integrity was severely disrupted by the upregulation of the inflammatory response (Fasano, 2011). All these previous findings supported the fact that intestinal tight junction and endotoxin were potential critical mediators during atherosclerosis development.

Sivelestat, with the formula of C_20_H_22_N_2_O_7_S, is one of the selective neutrophil elastase inhibitors for alleviating acute respiratory distress syndrome (Kawabata et al., 1991). For COVID-19 treatment, sivelestat also exhibited therapeutic benefits in recovering some patients with severe symptoms (Sahebnasagh et al., 2020). More recently, administration of sivelestat suppressed the endotoxin-induced neutrophil activity *in vitro* (Okeke et al., 2020). Multiple studies have supported excessive intestinal accumulation of neutrophil elastase; one of key neutrophil serine proteinases injured the mucosal structure and debilitated intestinal diseases, such as the thrombotic tendency (Maloy and Powrie, 2011; Li et al., 2020). Besides, neutrophil elastase exacerbated cardiovascular diseases (Warnatsch et al., 2015; Wen et al., 2018). Therefore, targeting neutrophil elastase by sivelestat had potential pharmacological benefits in improving the inflammatory response and related systemic diseases.

The present study aimed to utilize sivelestat to disclose the crosstalk between intestinal neutrophils and atherosclerotic development. Atherosclerotic Apo E^-/-^ mice were administered sivelestat, and we further investigated its pharmacological effects on atherosclerotic development and intestinal homeostasis. Our findings provided evidence that sivelestat was a potential drug to combat atherosclerosis, and the intestine/vascular interaction could explain the pharmacological effects of sivelestat on cardiovascular diseases.

## Materials and Methods

### Reagents

The Oil Red O staining kit (#MAK194), hematoxylin (#MHS32), eosin solution (#HT110116), sivelestat (#S7198), and lipopolysaccharides (#L2630) were purchased from Sigma (Sigma Chemicals, St. Louis). Anti-Ly6G antibody (#561105) was purchased from BD (BD Biosciences). Anti-moma-2 antibody (#ab33451) was purchased from Abcam (Cambridge, United Kingdom). Anti-ZO-1 (#5406), anti-Tubulin (#5568), anti-phosphorylated IκB (#2859), and anti-IκB (#9242) antibodies were purchased from Cell Signaling Technology, Inc. (Danvers, MA). DX-4000-FITC (#46944) was purchased from Sigma (Sigma chemicals, St. Louis), and the endotoxin kit (#88282) was from Pierce (Thermo Fisher, CA). Intestinal protein levels of TNF-α (#BMS607-3), IL-1β (#BMS6002), and MCP-1 (#BMS6005) were measured by using ELISA kits from Invitrogen (Thermo Fisher, CA). The intestinal neutrophil elastase activity was measured with a commercial kit (# ab204730) purchased from Abcam (Cambridge, United Kingdom).

### Treatment of Sivelestat *In Vivo*

All mouse experimental procedures were approved by the Animal Research and Teaching Committee at Southern Medical University (Guangzhou, China). The mice were housed in 21 ± 2 ^o^C condition and were given free access to diet and tap water. In detail, male aged 6-week ApoE-deficient mice (ApoE^-/-^) were purchased from the Shanghai Model Organisms Center (Shanghai, China), and every group included six mice. Mice were fed with high-fat high-cholesterol diet (HFHC, 40% fat and 1.25% cholesterol, Cat#D12108C, research diets). After being fed with HFHC for 4-week, the ApoE^-/-^ mice were administered with sivelestat (50 mg/kg per day, intraperitoneal injection) or phosphate-buffered saline (Veh) for another 8 weeks. For lipopolysaccharide stimulation, the ApoE^-/-^ mice were fed with HFHC for 4 weeks and then treated with sivelestat (50 mg/kg per day, intraperitoneal injection) and LPS (25 μg/day, subcutaneous injection) or Veh for 8 weeks. Then, mouse tissues, including the aorta, small intestine, and serum, were collected for further measurement.

### Measurement of Intestinal Permeability

For the analysis, 500 mg/kg FITC-labeled dextran was orally gavaged into mice, and the serum samples were collected for further analysis. The serum concentration of DX-4000-FITC was measured by using a fluorescence spectrophotometer (Synergy H1, BioTek, VT) with an excitation wavelength of 485 nm and an emission wavelength of 535 nm.

### Histological Staining of the Aorta and Intestine

For lipid staining of the en face aorta and aorta root, whole aorta or 10-µm frozen sections of the aorta root were stained with the Oil Red O staining kit. For hematoxylin and eosin staining, the aorta and colon were fixed in 4% paraformaldehyde and embedded in paraffin. Then, 5-µm paraffin sections were dehydrated and stained with hematoxylin and eosin solution. For immunofluorescence staining, 5-µm aorta sections were hydrated, blocked with 3% BSA solution, and incubated with the anti-moma-2 antibody at 1:100 dilution. After washing in PBS, the sections were stained with the fluorescence-labeled secondary antibody at 1:500 dilution. The nuclei were stained with DAPI. The images were viewed and captured by using a fluorescence microscope (Nikon, Tokyo), and relative lipid contents were measured in 50 images/per mouse by ImageJ software.

### Flow Cytometry Analysis of Intestinal Neutrophils

Intestinal resident cells were isolated from 5 to 8 cm of the intestine. Briefly, intestinal segments were excised, cleaned, and cut into small pieces. Samples were then washed with sterile bovine serum-free RPMI 1640 medium three times and digested in 10 ml RPMI containing 1 mg/ml collagenase A (Roche #10103578001) for 30 min at 37°C and 200 rpm in a shaking incubator. Digestion was quenched with FACS buffer and centrifuged for 5 min at 800 g. After washing with PBS t three times, the samples were passed through a 100-μm cell strainer to obtain a single cell suspension. For neutrophil staining, the cells were stained with anti-Cd11b and anti-Ly6G antibodies at 1:100 dilution. The cell percentage was analyzed by using a flow cytometer (BD FACSAria™ III).

### Culture of Rat Intestinal Epithelial Cells

The rat intestinal epithelial cell line (#IEC-18, ATCC) was gifted from Dr. Ning Zhu (Zhejiang University). Cells were cultured in DMEM containing 10% FBS and 1% penicillin/streptomycin antibiotics in a humidified chamber (37°C, 21% O_2_, and 5% CO_2_). For cell experiments, $1\times 10^{6}$ cells were pretreated with sivelestat (100 μM) or DMSO and stimulated with 0.1 μg/ml human neutrophil elastase recombinant protein for 24 h.

### Real-Time PCR Analysis of the Gene Expression

Total RNA from the whole aorta or intestine tissues was extracted by using TRIzol (Invitrogen), according to the manufacturer’s instructions. Complementary DNA was reverse-transcribed using the PrimeScript™ RT reagent kit with gDNA Eraser (Takara Biotech, Dalian, China), and qPCR was performed with the SYBR Green quantitative kit (Applied Biosystems, CA). The primer sequences used in this study were listed as follows: mouse *Zo-1*: F-5′-CAACATACAGTGACGCTTCACA-3’; R-5′- CAC​TAT​TGA​CGT​TTC​CCC​ACT​C-3′, mouse *Tnf-α*: F-5′-ACGGCATGGATCTCAAAGAC-3’ R-5′-AGATAGCAAATCGGCTGACG-3′, mouse *Il-1β*: F-5′-CTGGTGTGTGACGTTCCCATTA-3’; R-5′-CCGACAGCACGAGGCTTT-3′, mouse *Il-6*: F-5′-CCACGGCCTTCCCTAC-3’; R-5′- AAG​TGC​ATC​ATC​GTT​GT-3′, mouse *Mcp-1*: F-5′-CCACTCACCTGCTGCTACTCA-3’; R-5′-TGGTGATCCTCTTGTAGCTCTCC-3′, mouse *Vcam-1*: F-5′-CCGGCATATACGAGTGTGAA-3’; R-5′-TAGAGTGCAAGGAGTTCGGG-3′, and mouse *Gapdh*: F-5′-AGGAGCGAGACCCCACTAAC-3’; R-5′-GATGACCCTTTTGGCTCCAC-3’. The relative gene expression was calculated by normalizing to the *Gapdh* level.

### Immunoblot Analysis

Protein lysates were extracted from the intestinal tissues or epithelial cells, and the protein concentration was measured by using a BCA assay kit (Thermo Fisher, CA). 50 μg protein was subjected to 10% SDS–PAGE electrophoresis and electro-transferred to polyvinylidene difluoride membranes (Amersham Biosciences). Then, the membranes were blocked with 10% non-fat milk and incubated with anti-ZO-1, anti-phos-IκB, IκB, or anti-Tubulin antibody at 1:1,000 dilution and relative secondary antibodies at 1:5,000 dilution. The relative protein expression was visualized by using enhanced chemiluminescence reagents (Bio-Rad, CA) and quantitatively analyzed by ImageJ software.

### Clinical Study

This study included 26 individuals, including nine atherosclerotic subjects with carotid intima-medial thickness (IMT) ≥ 0.85 mm and 17 healthy subjects with IMT <0.85 mm, who were recruited from July 2019 to May 2020 at the Shenzhen Hospital of Southern Medical University (NYSZYYEC20190005). The basic clinical parameters of these subjects are showed in Supplementary Table S1. Furthermore, among these subjects, 12 patients were under intestinal polyp surgery (3 patients with ≥0.85 mm and nine subjects with IMT <0.85 mm). The subjects with IMT <0.85 mm were characterized with no history of angina and other heart diseases, a normal resting ECG, and normal exercise ECG stress testing. In patients under the intestinal polyp surgery, the intestinal activity was measured by using the commercial kit. Before statistical analysis, the Shapiro–Wilk test was conducted to identify the distribution for normality. Then, Pearson analysis was conducted for clinical correlation. All participants have been informed clinical consent, and the related analysis protocol was approved by the Human Ethics Committee of Southern Medical University.

### Statistical Analysis

Data were shown as mean ± SEM. The Student’s *t*-test was used for comparing two groups, and ANOVA was used for multiple groups (GraphPad, San Diego, CA). Pearson analysis was used for analyzing the statistical correlation. p < 0.05 was considered to be significant.

## Results

### Neutrophils Infiltrate Into the Intestine During the Atherosclerotic Process

**FIGURE 1:**
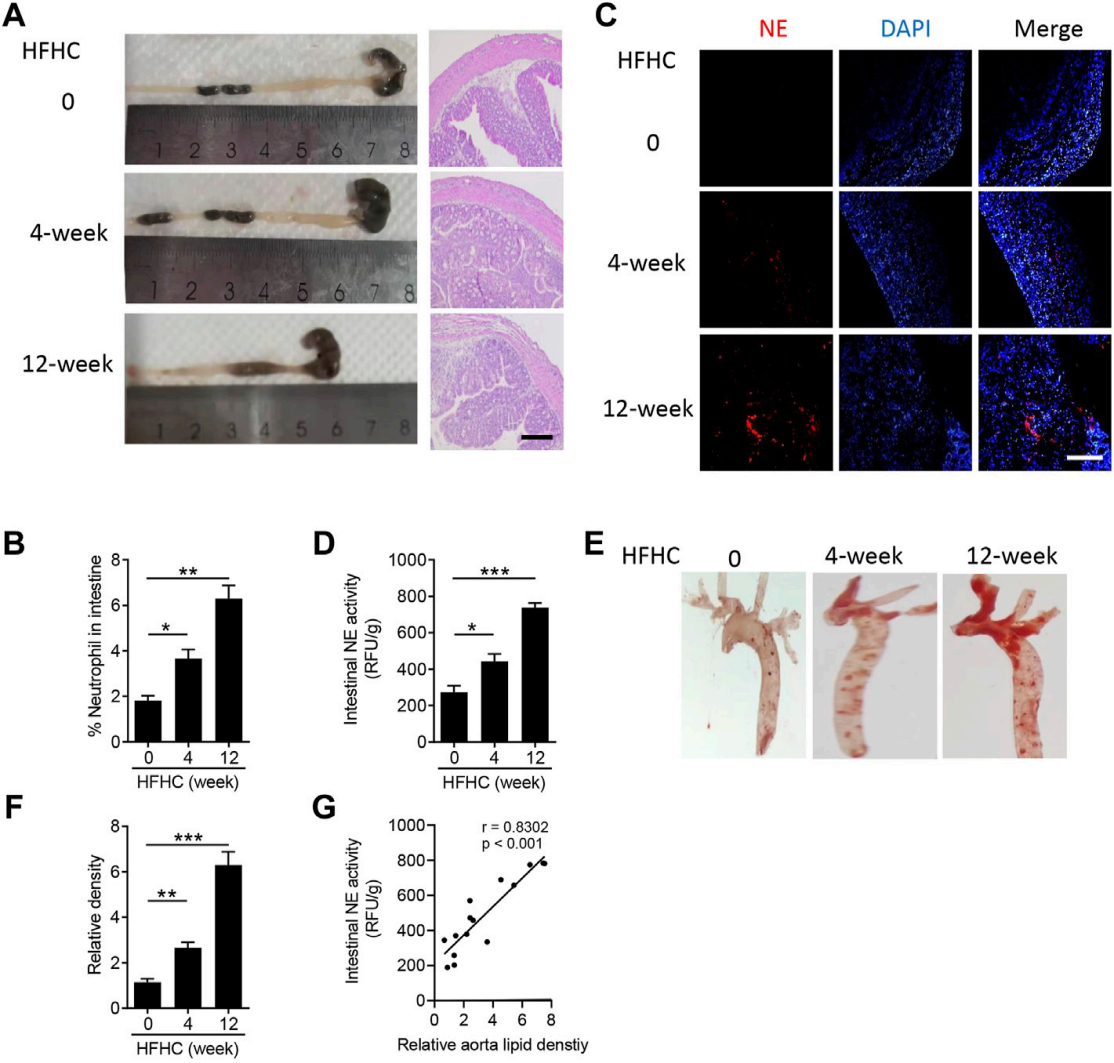
Neutrophils infiltrate into the intestine during the atherosclerotic process. Male Apo E^-/-^ mice were fed with high-fat high-cholesterol diet (HFHC) for 4 and 12 weeks, and ApoE^-/-^ mice were fed with normal diet as the control group. **(A)** Representative images of the mouse colon (left panel) and hematoxylin-eosin staining of the colon structure (right panel). Scale bar = 100 μm. **(B)** Flow cytometry analysis of the percentage of Cd11b^+^Ly6G^+^ neutrophils in the intestine. **(C)** Immunofluorescence staining of neutrophil elastase (NE, red color, neutrophil marker), and the nuclei were stained with DAPI (blue color). Scale bar = 100 μm. **(D)** Measurement of the neutrophil elastase (NE) activity in intestinal lysates. **(E,F)** Oil Red O staining of the en face aorta **(E)** and the quantitative analysis of the relative lipid density **(F)**. **(G)** Correlation of the intestinal NE activity and aorta lipid density. Data are shown as mean ± SEM. n = 5 mice/group, and *p < 0.05, **p < 0.01, ***p < 0.001.

Multiple studies have addressed high-fat diet severely injured intestinal homeostasis, such as intestinal shortening, disruption of the intestinal barrier, and inflammatory response (Araújo et al., 2017; Rohr et al., 2020). To identify the pathological changes of the intestine in atherosclerotic mice, at first, we measured the tissue remodeling of colon. As shown in Figure 1A, the length of the colon was significantly decreased after the mice were fed with high-fat high-cholesterol (HFHC) diet for 4 and 12 weeks, and HFHC feeding also induced colic structural disorders. For an inflammatory response, flow cytometry analysis indicated Cd11b^+^Ly6G^+^ neutrophils obviously infiltrated into the intestine (Figure 1B), especially in the HFHC-fed mice for 12 weeks (*p* < 0.01). Furthermore, neutrophil elastase (NE), one of the neutrophil serine proteases, was also highly expressed in the intestines of HFHC-fed mice (Figure 1C), and the intestinal NE activity was increased time-dependently during HFHC feeding (Figure 1D). Meanwhile, as shown in Figures 1E,F, mice with HFHC feeding had remarkable lipid plaque formation in the aorta. To testify the possible role of intestinal NE in atherosclerotic development, Pearson analysis showed the intestinal NE levels had a positive correlation of the aorta lipid deposit (Figure 1G).

### Administration of Sivelestat Attenuates HFHC Diet-Induced Atherosclerosis and Vascular Inflammation in ApoE^-/-^ Mice

**FIGURE 2:**
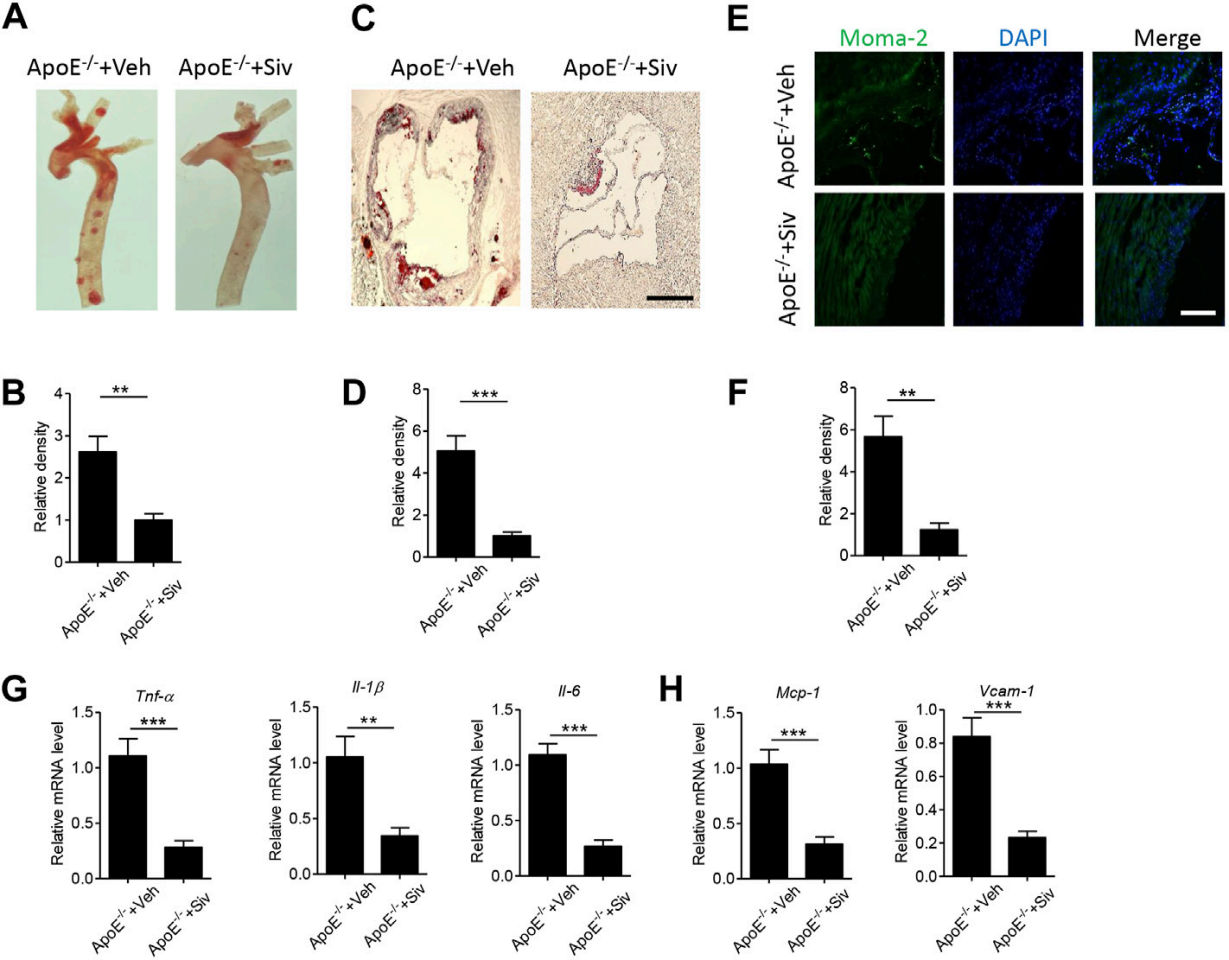
Sivelestat attenuates HFHC-induced atherosclerotic plaque formation and vascular inflammation in Apo E^-/-^ mice. Male Apo E^-/-^ mice were fed with HFHC diet for 4 weeks and then treated with sivelestat (Siv, 50 mg/kg per day, intraperitoneal injection) or phosphate-buffered saline (Veh) for 8 weeks. (A-B) Oil Red O staining of the en face aorta **(A)** and quantitative analysis of the relative lipid density **(B)**. **(C-D)** Oil red O staining of the aorta root **(C)** and quantitative analysis of the relative lipid density **(D)**. Scale bar = 200 μm. **(E-F)** Immunofluorescence staining of aorta root sections with anti-moma-2 antibody **(E)** and quantitative analysis of the relative density **(F)**. Scale bar = 100 μm. **(G-H)** Real-time PCR analysis of gene levels of inflammatory cytokines **(G)**, including *Tnf-α*, *Il-1β*, and *Il-6*, and chemokines **(H)** including *Mcp-1* and *Vcam-1*. Data are shown as mean ± SEM. n = 6 mice/group, and **p < 0.01 and ***p < 0.001.

Neutrophils play critical roles in the inflammatory response (Kolaczkowska and Kubes, 2013) and participated in the progress of cardiovascular diseases (Gaul et al., 2017). Sivelestat, as a selective inhibitor of neutrophil elastase, could suppress inflammation in several inflammatory diseases (Endo et al., 2006; Shimoda et al., 2008). In the present study, we aimed to explore the pharmacological effects of sivelestat on atherosclerosis in mice. Consistent with previous studies (Jin Li et al., 2016; Zhu et al., 2018), high-fat high-cholesterol (HFHC) diet accelerated lipid accumulation in the aorta from Apo E^-/-^ mice (Figures 2A–D). But the administration of sivelestat significantly decreased lipid contents in en-face aorta (Figures 2A,B, *p* < 0.01) and the sections of the aorta root (Figures 2C,D, *p* < 0.001). Infiltration of immune cells, including macrophages and other monocytes, was a key phenotype of atherosclerosis (Hansson, 2005). As shown in Figures 2E,F, treatment of sivelestat inhibited the infiltration of monocytes and macrophages (*p* < 0.01). Meanwhile, sivelestat also decreased the gene levels of inflammatory factors, including pro-inflammatory cytokines (Figure 2G, *p* < 0.01) and chemokines (Figure 2H, *p* < 0.001). However, sivelestat had no significant effects on the other basic parameters of a mouse, including body weight, the fasting blood glucose level, and serum lipid profiles (Supplementary Table S2).

### Administration of Sivelestat Decreases Endotoxemia by Improving Zonula Occludens-1-Mediated Intestinal Permeability and Intestinal Inflammatory Response *In Vivo* and *In Vitro*

**FIGURE 3:**
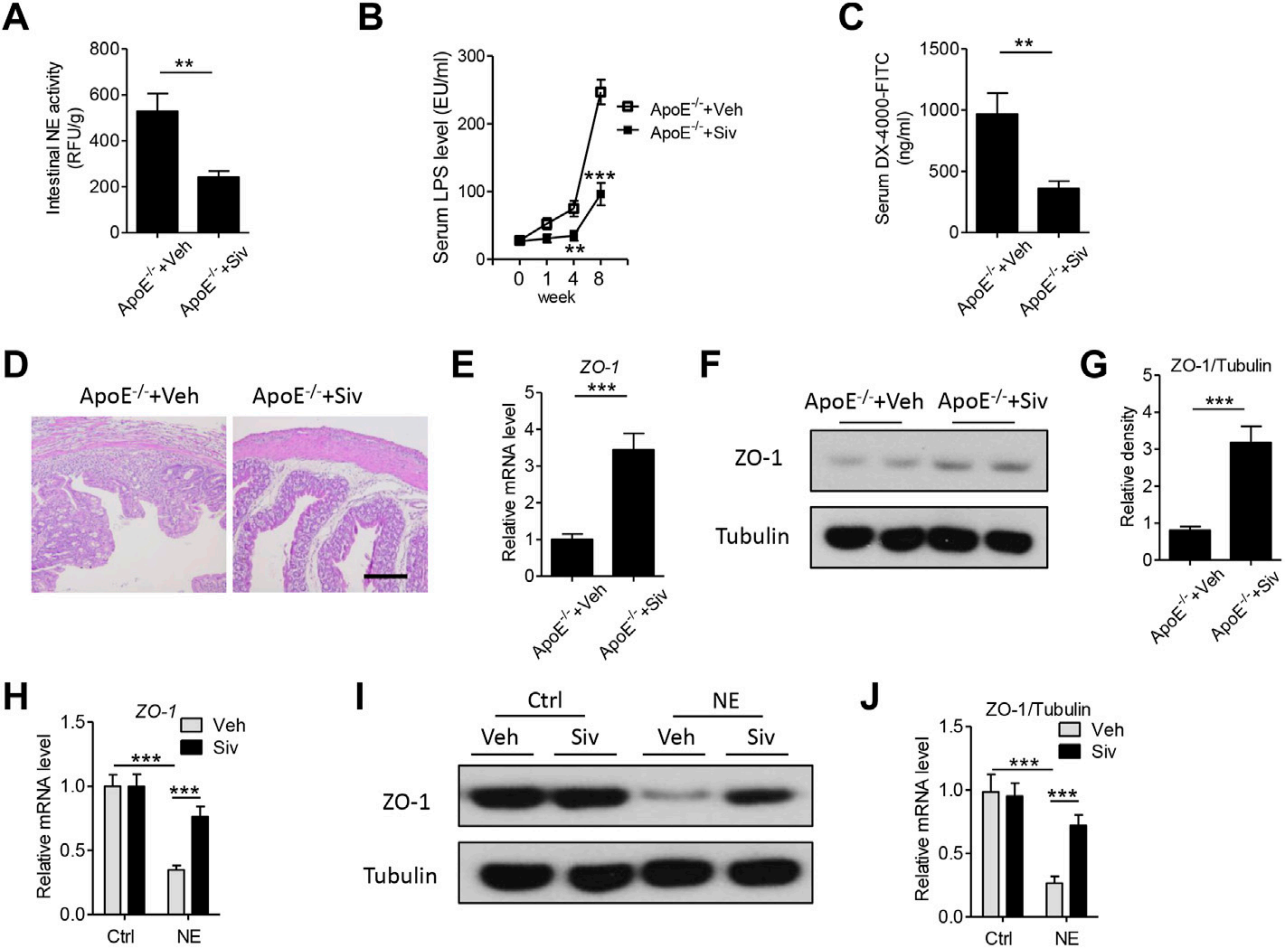
Sivelestat decreases HFHC-induced endotoxemia and intestinal structural disorders by upregulating the intestinal zonula occludens-1 expression in Apo E^-/-^ mice and intestinal epithelial cells. **(A–F)** Male Apo E^-/-^ mice were fed with HFHC diet for 4 weeks and then treated with sivelestat (Siv, 50 mg/kg per day, intraperitoneal injection) or Veh for 8 weeks. **(A)** Mouse intestinal NE activity. **(B)** Measurement of circulating lipopolysaccharide (LPS) levels after sivelestat treatment for 0, 1, 4, and 8 weeks. **(C)** Mice were orally administered with FITC-labeled dextran, and the circulating concentration of DX-4000-FITC was analyzed. **(D)** Hematoxylin and eosin (HE) staining of the colon. Scale bar = 100 μm. **(E)** Gene expression of intestinal zonula occludens (ZO)-*1*. **(F–G)** Immunoblot analysis of intestinal ZO-1 **(F)** and quantitative analysis of the relative density of ZO-1/Tubulin **(G)**. **(H–J)** $1\times 10^{6}$ rat intestinal epithelial cells were pretreated with sivelestat (100 μM) or DMSO and stimulated with 0.1 μg/ml human neutrophil elastase recombinant protein for 24 h. **(H)** Real-time PCR analysis of the ZO-1 gene level. **(I–J)** Immunoblot analysis of ZO-1 protein expression **(I)** and quantitative analysis of the relative density of ZO-1/Tubulin **(J)**. Data are shown as mean ± SEM. n = 6 mice/group or n = 5 independent experiments, and **p < 0.01, ***p < 0.001.

The abnormal induction of intestinal permeability and subsequent endotoxemia could initiate the progress of atherosclerotic plaque formation (Zhu et al., 2018). In the present study, we found the treatment of sivelestat led to a significant reduction in the intestinal NE activity (Figure 3A) and the circulating level of endotoxin in the HFHC-fed ApoE^-/-^ mice (Figure 3B). Next, we measured intestinal permeability by treating mice with fluorescent-labeled dextran (DX-4000-FITC). As shown in Figure 3C, the circulating concentration of DX-4000-FITC was decreased in sivelestat-treated Apo E^-/-^ mice (p < 0.01). H&E staining of the colon also showed the HFHC diet loosened the structure of mucosa, which facilitated the leakage of toxic substances into circulation, whereas sivelestat improved the intestinal structure (Figure 3D). Intestinal permeability was mainly controlled by zonula occludens (ZO)-1, one of the key epithelial tight junction proteins (Van Itallie et al., 2009). As shown in Figure 3E, the intestinal gene level of ZO-1 was upregulated in sivelestat-treated Apo E^-/-^ mice (p < 0.001). The protein expression of ZO-1 was also significantly upregulated in sivelestat-treated Apo E^-/-^ mice, as compared with Veh-treated Apo E^-/-^ mice (Figures 3F,G). To determine the direct pharmacological effects of sivelestat on the ZO-1 protein expression, intestinal epithelial cells were treated with NE recombinant protein with or without sivelestat. Figures 3H–J showed NE recombinant protein decreased the gene and protein expression of ZO-1 (p < 0.001), whereas sivelestat attenuated NE-induced ZO-1 reduction in intestinal epithelial cells (p < 0.05).

**FIGURE 4:**
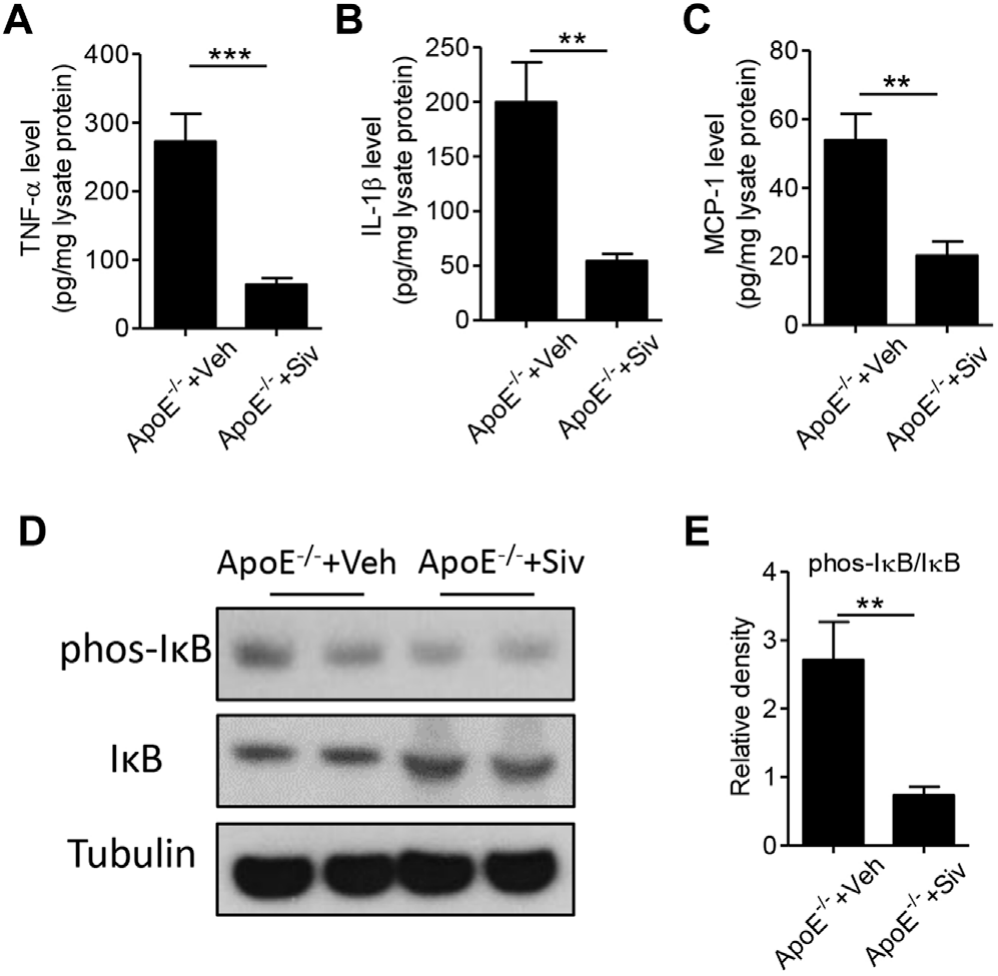
Sivelestat attenuates intestinal inflammation in HFHC-fed Apo E^-/-^ mice and NE-treated intestinal epithelial cells. **(A–E)** Male Apo E^-/-^ mice were fed with HFHC diet for 4 weeks and then treated with sivelestat (Siv, 50 mg/kg per day, intraperitoneal injection) or Veh for 8 weeks. ELISA analysis of intestinal protein levels of TNF-α **(A)**, IL-1β **(B)**, and MCP-1 **(C)**. Immunoblot analysis of protein expressions of phosphorylated (phos-) IκB and IκB **(D)** and quantitative analysis of the relative density of phos-IκB/IκB **(E)**. Data are shown as mean ± SEM. n = 6 mice/group, and **p < 0.01 and ***p < 0.001.

Previous studies have showed there were abnormal immune responses in the gastrointestinal homeostasis of patients with atherosclerosis (Cani et al., 2007; Pendyala et al., 2012). Therefore, we investigated the effects of sivelestat on the intestinal inflammatory response in the HFHC-fed Apo E^-/-^ mice. As shown in Figures 4A–C, the protein levels of inflammatory cytokines, including TNF-α (Figure 4A, p < 0.001), IL-1β (Figure 4B, *p* < 0.01), and MCP-1 (Figure 4C, p < 0.01), were significantly decreased in intestinal lysates from sivelestat-treated ApoE^-/-^ mice. NF-κB signaling is one of the key transcriptional factors in regulating the inflammatory response (Pan et al., 2012; Pan et al., 2014). To this end, we measured the expression of phosphorylated (phos-) IκB and IκB in intestinal tissues. Figures 4D,E showed sivelestat inhibited NF-κB activation by decreasing phos-IκB and increasing the IκB level (*p* < 0.01).

### Administration of Lipopolysaccharides Eliminates the Anti-Atherosclerotic Benefits of Sivelestat in HFHC-Fed ApoE^-/-^ Mice

**FIGURE 5:**
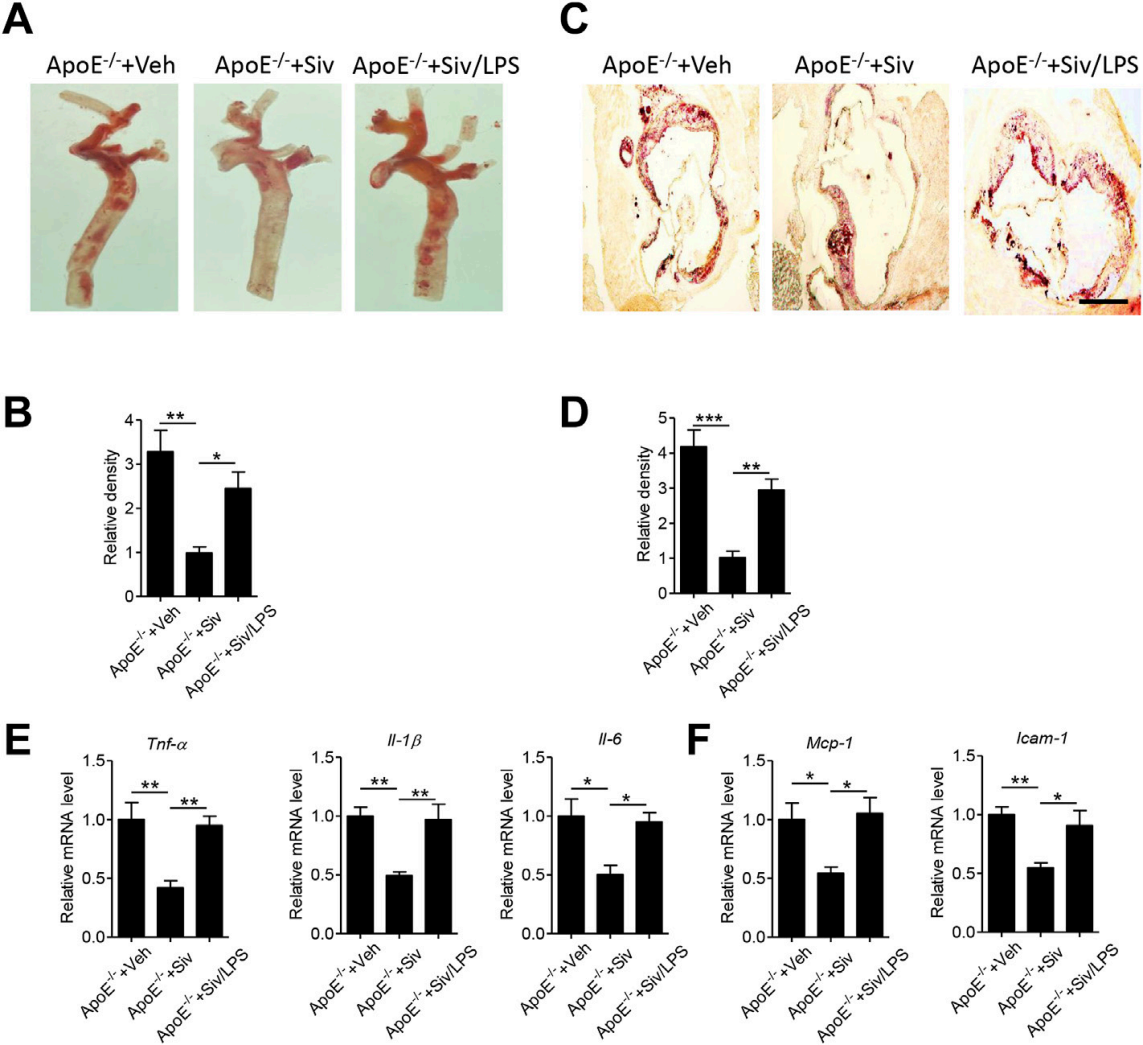
Administration of lipopolysaccharides eliminates anti-atherosclerotic benefits of sivelestat in Apo E^-/-^ mice. Male Apo E^-/-^ mice were fed with HFHC diet for 4 weeks and then treated with sivelestat (Siv, 50 mg/kg per day, intraperitoneal injection) and LPS (25 μg/day, subcutaneous injection) or Veh for 8 weeks. **(A–B)** Oil Red O staining of en face aorta **(A)** and the quantitative analysis of relative lipid density **(B)**. **(C–D)** Oil red O staining of the aorta root **(C)** and the quantitative analysis of the relative lipid density **(D)**. Scale bar = 200 μm. **(E–F)** Real-time PCR analysis of gene levels of inflammatory cytokines **(E)**, including *Tnf-α*, *Il-1β*, and *Il-6*, and chemokines **(F)** including *Mcp-1* and *Vcam-1*. Data are shown as mean ± SEM. n = 6 mice/group and *p < 0.05, **p < 0.01, ***p < 0.001.

To clarify whether the pharmacological benefits of sivelestat is dependent on lowering endotoxemia, we subcutaneously injected lipopolysaccharides (LPS) into sivelestat-treated ApoE^-/-^ mice. Administration of sivelestat decreased lipid accumulation in the aorta of Apo E^-/-^ mice (Figures 1A–D), but co-treatment with LPS reversely increased lipid contents in the en-face aorta (Figures 5A,B, *p* < 0.05) and the sections of the aorta root (Figures 5C,D, *p* < 0.01). Real-time PCR analysis of the mouse aorta further showed LPS eliminated the benefits of sivelestat on suppression of vascular inflammation, characterized by reduction of pro-inflammatory cytokines and chemokines (Figures 5E,F, *p* < 0.05).

### Circulating Endotoxin Level and Intestinal NE Activity Were Potential Clinical Diagnostic Biomarkers of Atherosclerosis

**FIGURE 6:**
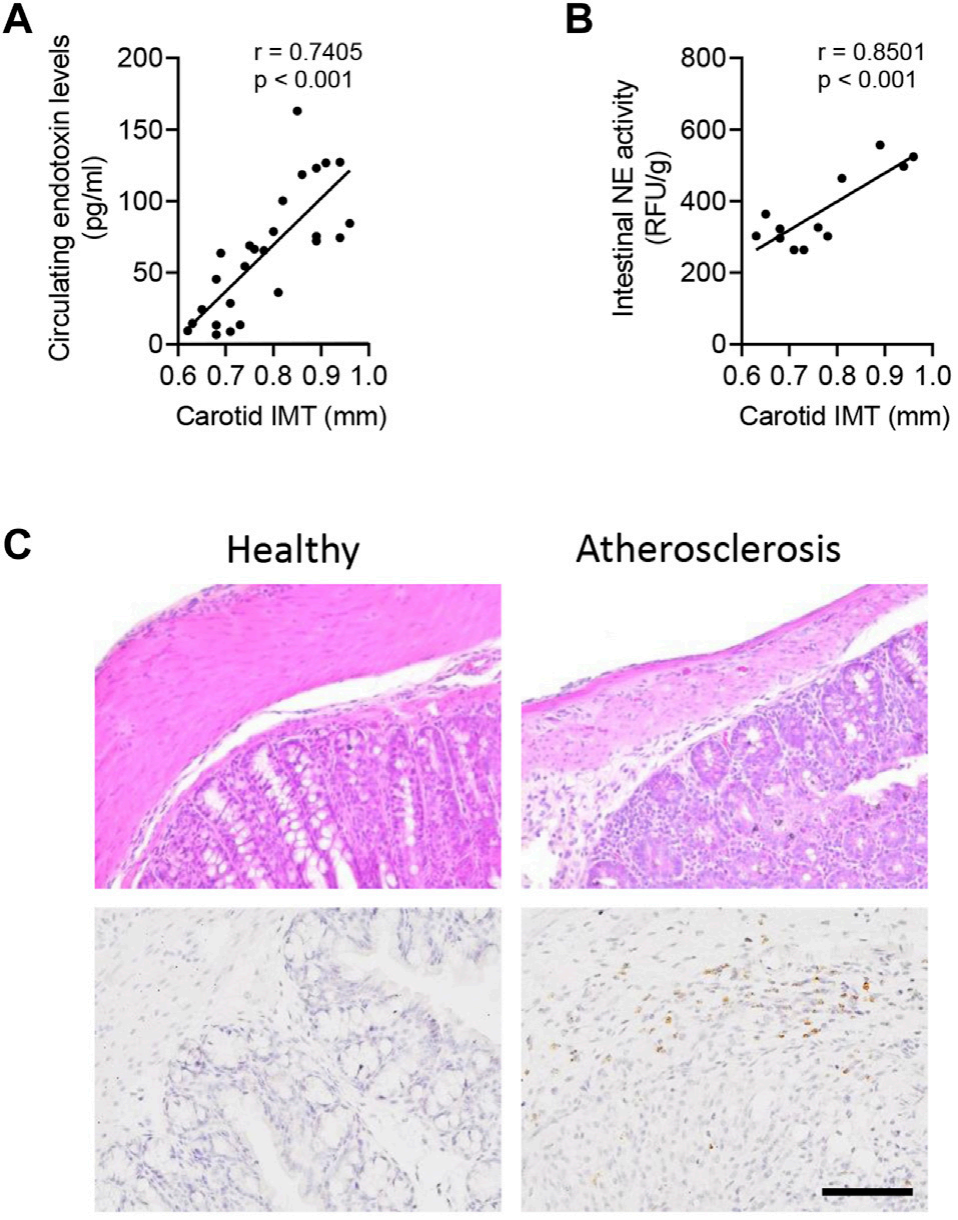
Intestinal NE level is closely associated with the human atherosclerotic status. In total, 26 volunteers were recruited, including 12 patients with intestinal polyp surgery. **(A)** Correlation between circulating levels of endotoxin and carotid intima-medial thickness (IMT). **(B)** Measurement of intestinal NE activity in 12 patients with intestinal polyp surgery and further correlation of the intestinal NE activity and carotid IMT. **(C)** HE staining and immunohistological staining of NE in human intestines.

To address the clinical applications, 26 volunteers were recruited for atherosclerotic analysis, including 12 patients with intestinal polyp surgery. The circulating endotoxin level and carotid intima-medial thickness (IMT) were measured in all subjects (Supplementary Table S1). As shown in Pearson analysis, the carotid IMT value was positively associated with the circulating level of endotoxin (Figure 6A) and intestinal NE activity (Figure 6B). Furthermore, there were structural disorders and higher expression of NE in atherosclerotic patients, as compared with healthy subjects (Figure 6C).

## Discussion

Emerging studies have demonstrated modulation of intestinal homeostasis as one of the potential approaches for protection against atherosclerosis (Jin Li et al., 2016; Zhu et al., 2018). In patients with atherosclerosis, there was excessive inflammation and disruption of intestinal permeability, characterized by disorders of tight junction (Chuanwei Li et al., 2016). By utilizing sivelestat as a research tool, the present study identified that neutrophil elastase, one of the essential inflammatory mediators, contributed to atherosclerotic plaque formation. Administration of sivelestat attenuated diet-induced aorta plaque formation and vascular inflammation, accompanied by lowering endotoxemia. Mechanistically, sivelestat improved diet- or recombinant neutrophil elastase protein-induced intestinal permeability by upregulation of zonula occludens-1 and inhibited the intestinal inflammatory response. However, replenishment of lipopolysaccharides eliminated the anti-atherosclerotic benefits of sivelestat in mice. Pearson analysis of clinical parameters further supported circulating endotoxin and intestinal NE, which were potential diagnostic biomarkers of atherosclerotic patients.

The crosstalk between gastrointestinal homeostasis and cardiovascular disease is an attractive topic in recent years. Intestinal metabolites, such as TMAO, promoted atherosclerotic plaque formation in humans and mice (Koeth et al., 2013). Moreover, TMAO activated the cardiac autonomic nervous system and deteriorated ischemia-induced ventricular arrhythmia (Meng et al., 2019). Intestinally derived lysophosphatidic acid accelerated the atherosclerotic process dependent on hyperlipidemia and excessive inflammatory response (Navab et al., 2015). In addition, the incidence of cardiovascular diseases was closely associated with the component of intestinal microbiota. Administration of beneficial microbial species, such as *Akkermansia muciniphila*, improved Western diet-induced atherosclerosis in atherosclerotic Apo E^-/-^ mice (Jin Li et al., 2016). In contrast, one microbial component named *Citrobacter*, a species of toxic bacteria, was positively correlated with carotid intima-media thickness in a Bangladesh population (Wu et al., 2019).

Furthermore, intestinal homeostasis also depends on the structural maintenance of the intestinal epithelium. A higher level of circulating endotoxin, as a consequence of abnormal intestinal leakage, contributed to the development of multiple diseases. Abnormal intestinal permeability and the consequence of endotoxemia were some key characteristics in patients with fatty liver diseases (Parlesak et al., 2000). In atherosclerotic mice, it was observed that severe abruption of intestinal permeability and endotoxemia but improvement of intestinal homeostasis could attenuate atherosclerotic plaque formation and vascular inflammation (Jin Li et al., 2016; Zhu et al., 2018). Mechanistically, tight junction proteins, such as zonula occludens-1 (ZO-1) and occludin, determined the intestinal structure and permeability (Van Itallie et al., 2009; Hamada et al., 2010). The expression profile of ZO-1 was a key biomarker of atherosclerotic development, whereas upregulation of ZO-1 could improve intestinal permeability and vascular plaque formation (Zhang et al., 2020). Moreover, a variety of studies have demonstrated that the transcriptional biogenesis and activity of ZO-1 were closely associated with the intestinal inflammatory response. Chronic, excessive inflammation injured the tight junction barrier in multiple diseases (Fasano, 2011). Suppression of intestinal inflammation, such as inhibition of nuclear factor (NF)-κB signaling, was one of the therapeutic approaches to improve intestinal homeostasis and consequent disorders (Arakawa et al., 2019; Nunes et al., 2019). Consistently, the present study also found the intestinal ZO-1 expression was decreased in atherosclerotic mice but induction of ZO-1 concurred in sivelestat-treated Apo E^-/-^ mice. Meanwhile, administration of sivelestat decreased the diet-induced excessive expression of intestinal inflammatory cytokines and NF-κB activity.

Neutrophils, as one of the early pro-inflammatory cells, have been shown to affect plaque formation and plaque rupture (Zernecke et al., 2008; Ionita et al., 2010). Due to their relatively short lifespan, neutrophils are rarely detected in atherosclerotic lesions (Nathan, 2006; Galli et al., 2011). Emerging studies have demonstrated that neutrophils may exacerbate cardiovascular diseases through the release of neutrophil serine proteinases, thus inducing atherosclerotic plaque formation (Warnatsch et al., 2015; Wen et al., 2018). More recently, Wen et al. (2018), reported pharmacologic inhibitors of neutrophil elastase could improve the atherosclerotic process in Apo E^-/-^ mice. Okeke et al. (2020), interestingly, found suppression of neutrophil elastase rescued mice from endotoxic shock, which indicated the close links between the neutrophil elastase activity and endotoxemia. However, there was no direct evidence providing the roles of neutrophil elastase in mediating intestine/vascular crosstalk.

Sivelestat, with the formula C20H22N2O7S, is a selective neutrophil elastase inhibitor for alleviating acute respiratory distress syndrome (Kawabata et al., 1991). In a lipopolysaccharide-induced septic mouse model, sivelestat attenuated alveolar collapse and stromal tissue thickening (Inoue et al., 2005). More recently, administration of sivelestat suppressed NET formation *in vitro* but rescued mice from lipopolysaccharide-induced endotoxic shock (Okeke et al., 2020). However, there was no report to disclose the pharmacological effects of sivelestat in atherosclerotic formation and vascular inflammation. Our present findings, for the first time, uncovered the anti-atherosclerotic effects of sivelestat in genetic Apo E^-/-^ mice; then, we determined the intestine/vascular axis was a potential explanation for the therapeutic benefits of sivelestat *in vivo* and *in vitro*.

In conclusion, the present findings supported intestinal NE-regulated intestinal permeability and inflammatory response in the development of atherosclerosis, and administration of sivelestat had benefits in protection against atherosclerosis.

## Data Availability

The raw data supporting the conclusion of this article will be made available by the authors, without undue reservation.
