# Supplementary material for: Sivelestat Alleviates Atherosclerosis by Improving Intestinal Barrier Function and Reducing Endotoxemia
Source: Front Pharmacol. 2022 Apr 4;13:838688. doi: 10.3389/fphar.2022.838688 (PMC9014170; doi:10.3389/fphar.2022.838688)
Supplement: Supplementary file 1 [file DataSheet1.docx]

**Table S1. The basic clinical parameters of recruited subjects.**

| Parameters/group | Healthy | Atherosclerosis | p-value |
| --- | --- | --- | --- |
| Gender (male/female) | 4/5 | 11/6 |  |
| Age | 43.32 ± 15.42 | 47.51 ± 11.95 | 0.447 |
| IMT (mm) | 0.73 ± 0.10 | 0.92 ± 0.06 | <0.001 |
| Endotoxin (pg/ml) | 41.12 ± 19.83 | 107.17 ± 26.83 | <0.001 |
| Body weight (kg) | 67.14 ± 24.55 | 76.66 ± 21.34 | 0.314 |
| Fasting blood glucose (mmol/L) | 4.42 ± 0.41 | 4.92 ± 0.96 | 0.128 |
| Plasma triglyceride (mmol/L) | 0.72 ± 0.21 | 1.06 ± 0.51 | 0.099 |
| Plasma cholesterol (mmol/L) | 4.73 ± 1.21 | 6.62 ± 1.56 | 0.003 |
| Plasma HDL-cholesterol (mmol/L) | 1.13 ± 0.25 | 0.71 ± 0.32 | 0.002 |
| Plasma LDL-cholesterol (mmol/L) | 3.77 ± 0.41 | 5.71 ± 1.02 | <0.001 |
| LDL/HDL ratio | 2.53 ± 0.54 | 4.82 ± 1.54 | <0.001 |

**Table S2. The lipid profiles of mice treated with sivelestat.**

| Parameters/group | ApoE^-/-^+Veh | ApoE^-/-^+Siv | p-value |
| --- | --- | --- | --- |
| Body weight (g) | 34.42 ± 5.46 | 32.80 ± 4.82 | 0.599 |
| Fasting blood glucose (mmol/L) | 6.52 ± 0.61 | 6.22 ± 0.43 | 0.332 |
| Serum triglyceride (mg/dL) | 2.38 ± 0.50 | 3.02 ± 0.63 | 0.079 |
| Serum cholesterol (mg/dL) | 17.68 ± 1.96 | 16.35 ± 2.65 | 0.346 |
| Serum HDL-cholesterol (mg/dL) | 3.13 ± 0.25 | 3.30 ± 0.30 | 0.119 |
| Serum LDL-cholesterol (mg/dL) | 14.32 ± 2.6 | 13.52 ± 2.23 | 0.579 |
